# Hydration Strategies and Body Composition Differences in Male and Female Elite Bodybuilders During Competition

**DOI:** 10.3390/nu17091554

**Published:** 2025-04-30

**Authors:** Frano Giakoni-Ramírez, Catalina Muñoz-Strale, Josivaldo de Souza-Lima, Luis Aránguiz Dote, José Francisco López-Gil, Vicente Javier Clemente-Suárez, Rodrigo Yáñez-Sepúlveda

**Affiliations:** 1 Faculty of Education and Social Sciences, Institute Sports and Wellbeing, Universidad Andres Bello, Las Condes, Santiago 7550000, Chile; frano.giakoni@unab.cl (F.G.-R.); catalina.munoz@unab.cl (C.M.-S.); llaranguizdote@uandresbello.edu (L.A.D.); 2 One Health Research Group, Universidad de Las Américas, Quito 170124, Ecuador; 3 Faculty of Sports Sciences, Universidad Europea de Madrid, Tajo Street, s/n, 28670 Madrid, Spain; vctxente@yahoo.es; 4 Grupo de Investigación en Cultura, Educación y Sociedad, Universidad de la Costa, Barranquilla 080002, Colombia; 5 Faculty of Education and Social Sciences, Institute Sports and Wellbeing, Universidad Andres Bello, Viña del Mar 2520000, Chile; rodrigo.yanez.s@unab.cl

**Keywords:** bodybuilding, bioelectrical impedance analysis, phase angle, hydration status, body composition, intracellular water, extracellular water

## Abstract

**Background:** Body composition and hydration status, particularly the balance between intracellular (ICW) and extracellular (ECW) water compartments, are critical factors influencing performance and aesthetics in competitive bodybuilding. Despite their significance, limited research has explored sex-based differences in hydration strategies and their impact on competitive outcomes. **Objectives**: This study aimed to characterize and compare ICW and ECW distribution, as well as their relationship with key physiological parameters, between male and female elite bodybuilders during an international competition. **Methods**: A total of 34 elite bodybuilders (18 males and 16 females) participated in this cross-sectional study. Body composition was assessed using multifrequency bioelectrical impedance analysis (BIA). The parameters evaluated included total body water, ICW, ECW, and phase angle (PhA). Differences between sexes were analyzed using Student’s *t*-tests, with statistical significance set at *p* < 0.05. **Results**: Significant sex-based differences were observed in water compartment distribution. Male bodybuilders exhibited higher ICW values (33.1 ± 2.8 L vs. 25.7 ± 2.5 L; *p* < 0.001) and PhA (8.2 ± 0.7 vs. 7.0 ± 0.9; *p* < 0.05), indicating greater muscle mass and cell integrity. In contrast, females had a higher ECW-to-total body water ratio (42.7% vs. 39.5%; *p* < 0.05), likely influenced by hormonal and metabolic factors. These findings suggest that sex-specific physiological characteristics should be considered when developing hydration and competition strategies. **Conclusions**: The study highlights the importance of sex-specific approaches to hydration and body composition management in bodybuilding. The observed differences in ICW and ECW distributions underscore the need for individualized training, nutrition, and hydration strategies to optimize competitive performance while maintaining athlete health. Future research should focus on longitudinal assessments to better understand hydration dynamics across different stages of competition preparation.

## 1. Introduction

Body composition and hydration levels are critical for athletic performance and health in bodybuilding, where aesthetics, symmetry, and muscle definition are key evaluation criteria [1]. Competitive success relies on maximizing muscle mass while minimizing body fat to optimize the physique’s visual presentation [2]. A higher intracellular-to-extracellular water (ICW/ECW) ratio enhances muscle definition and fullness, whereas fluid and carbohydrate manipulation strategies are used to achieve a “dry” appearance before competitions [3,4]. Proper management of these strategies is crucial, as errors can impact aesthetics and performance. ICW, essential for muscle function, protein synthesis, and cell signaling, contributes to muscle size and vascularity [5,6,7,8,9]. Conversely, excess ECW can obscure muscle definition, while excessive reduction may impair nutrient transport and muscle recovery [10,11]. Recent studies using BIVA techniques have shown promise in overcoming limitations of traditional BIA methods in physique athletes [12]; there is still a need to establish specific baseline values for intracellular and extracellular water distribution in elite bodybuilders during competition A more in-depth exploration of this aspect would facilitate the refinement of hydration strategies tailored to physique sports, a field that is currently deficient in empirical data, and would provide valuable insights to enhance competitive preparation.

The balance between intracellular and extracellular water is crucial for optimizing both muscle aesthetics and physical performance in bodybuilders [13]. However, extreme water manipulation strategies, such as forced dehydration, can disrupt this balance, leading to adverse effects like reduced muscle strength, fatigue, and increased injury risk [14]. Maintaining optimal hydration not only enhances physical appearance but also supports post-training recovery, emphasizing the need for proper ICW and ECW management [3]. During peak week (the final week before competition, when athletes fine-tune their physique through dietary and hydration adjustments), hydration strategies should be carefully monitored to prevent health and performance issues [15]. Evidence-based protocols are essential to maximize aesthetic and functional benefits while safeguarding athlete well-being. ICW and ECW balance can be effectively assessed through phase angle (PhA), a noninvasive marker obtained via bioelectrical impedance analysis (BIA), which reflects cell membrane integrity and tissue functionality [16,17]. A higher PhA indicates a favorable ICW/ECW ratio, crucial for maximizing muscle hypertrophy, minimizing catabolism, and optimizing aesthetics and performance [18]. Moreover, elevated PhA values are linked to improved muscle recovery and reduced fatigue, which are essential during high-intensity training [19].

Phase angle is not only correlated with cell volume but also serves as an indicator of overall muscle tissue quality, offering a comprehensive assessment of an athlete’s physical condition [20]. Its sensitivity to detecting subtle changes in body composition makes it a valuable tool for evaluating the real-time impact of nutritional and hydration strategies [21]. Various factors influence PhA in bodybuilding, including muscle mass, hydration protocols, and training regimens implemented during competition preparation [22]. Additionally, sex-based differences in PhA have been observed, highlighting physiological variability and distinct responses to training and fluid manipulation [12,23]. These differences may be influenced by hormonal factors, differences in muscle composition, and metabolic responses, which warrant further investigation to better understand their implications for competitive preparation.

In the context of bodybuilding, the continuous monitoring of PhA allows athletes and coaches to fine-tune hydration strategies, ensuring an optimal balance between intracellular and extracellular water to maximize muscle definition while maintaining functional performance. This monitoring becomes particularly crucial during peak week—the final stage before competition—where even minor fluctuations can significantly impact the athlete’s appearance and presentation [15]. As such, PhA has become an indispensable tool for optimizing both body composition and hydration management, reinforcing its importance in peak week planning and other key phases of competition preparation [4].

Competitive bodybuilding involves intense training, strict dietary control, and extreme body composition manipulations to achieve both optimal performance and aesthetic presentation on stage [7]. Unlike endurance and team sports, bodybuilding presents unique demands that require specialized approaches, particularly in the management of body fat and muscle mass [11,24,25]. Biological and hormonal differences between men and women lead to variations in lean mass distribution, fat levels, and hydration status, which influence training, recovery, and competition strategies [26,27]. These distinctions highlight the need for individualized training and nutrition programs tailored to the specific physiological characteristics of each athlete, an area that remains underexplored in the literature. Despite the growing interest in bodybuilding science, detailed research on body composition and hydration remains limited, particularly regarding the precise manipulation of these variables for competitive success [9]. Many previous studies relied on indirect or less accurate assessment methods, reducing result reliability [28]. Advanced techniques, such as multifrequency bioelectrical impedance analysis (BIA), provide a noninvasive, accurate evaluation of lean mass, fat mass, and water compartments, offering a comprehensive view of athlete fitness and enabling real-time adjustments to optimize competition preparation [17,22].

Despite the growing popularity and professionalization of bodybuilding, research on body composition and hydration management in competitive settings remains limited. The balance between intracellular and extracellular water is critical for optimizing muscle aesthetics, performance, and recovery, yet improper hydration strategies can pose significant health risks. Understanding sex-based differences in water distribution and their impact on functional parameters is essential to develop evidence-based preparation strategies that enhance competitive outcomes while ensuring athlete safety. Therefore, the objective of this study was to analyze and compare ICW and ECW distribution and their relationship with key functional parameters in elite male and female bodybuilders during an international competition. The findings aim to provide a scientific basis for personalized training, nutrition, and hydration protocols, contributing to safer and more effective practices in sports areas. It was hypothesized that male bodybuilders would exhibit a higher proportion of ICW relative to total body water compared to female competitors, due to their greater muscle mass and cell volume, while females are expected to present a higher relative proportion of ECW, potentially influenced by hormonal and physiological factors affecting fluid distribution and retention.

## 2. Materials and Methods

### 2.1. Participants

Participants in this study were selected using a convenience sampling method during the Mr. Universe Championship held in Santiago, Chile. Athletes voluntarily participated in the study after meeting the following inclusion criteria: (1) being over 18 years old, (2) being a registered member of their respective national federations, and (3) having successfully passed the official weigh-in of the event. A total of 34 elite bodybuilders, comprising 16 females (aged 39.2 ± 10.1 years, with 10.8 ± 10.0 years of competitive experience) and 18 males (aged 32.1 ± 7.6 years, with 8.0 ± 6.1 years of competitive experience), were enrolled after providing written informed consent. Participants represented several countries, including Brazil (*n* = 1), Mexico (*n* = 1), Nicaragua (*n* = 1), Ecuador (*n* = 2), Peru (*n* = 3), Venezuela (*n* = 3), Argentina (*n* = 6), and Chile (*n* = 16). Prior to weigh-in, athletes self-reported an average fluid intake of 144 mL within the last 24 h. Carbohydrate loading typically lasts 1 to 3 days, with intake reaching up to 8–10 g/kg of body weight in some cases. In the days prior, some athletes reduce carbohydrate intake to as low as 1–2 g/kg to deplete glycogen stores and trigger a supercompensation effect. Protein intake remains stable or slightly increases throughout the week to preserve lean muscle mass. Fat consumption is generally kept low to allow greater caloric flexibility for carbohydrates and protein. While male bodybuilders tend to consume more in total due to greater body mass, the overall nutritional strategy during peak week is similar between men and women. The averages for the competition week can be seen in Table 1.

The study was conducted in accordance with the ethical principles outlined in the Declaration of Helsinki [29]. The protocol was approved by the Scientific Ethics Committee of the Universidad Viña del Mar (Code R62-19b, approved on 17 December 2019).

### 2.2. Measurements

To ensure methodological clarity, this section outlines the parameters assessed and the techniques used for data collection. The measurements included anthropometric variables, body composition assessments, and hydration status, obtained using standardized procedures. Data collection was conducted by a team of 2 trained professionals, who underwent standardized training in bioelectrical impedance analysis and anthropometric assessments to ensure consistency and minimize measurement variability.

#### 2.2.1. Anthropometric Measurements

Body weight (kg) and height (cm) were measured using a calibrated stadiometer and digital scale (Seca 220, Hamburg, Germany) with a precision of 0.1 kg and 0.1 cm, respectively. Body mass index (BMI) was calculated as body weight divided by height squared (kg/m^2^), following the standards established by the World Health Organization (WHO).

#### 2.2.2. Bioelectrical Impedance Analysis (BIA)

Body composition measurements were performed via a multifrequency bioelectrical impedance analyzer, InBody 970 (InBody, Seoul, Republic of Korea), which has good specificity for assessing adults [30]. The device was calibrated before each session according to the manufacturer’s instructions, and all measurements were conducted by trained professionals to ensure consistency. The validity and reliability of BIA in assessing body composition have been confirmed in previous research [31]. The participants stood barefoot and in competition attire, ensuring direct contact between the palms of their hands and the soles of their feet with the device’s electrodes. During the measurement, which lasted approximately 60 s, the participants remained still and silent, with their arms slightly separated from their torso. The parameters evaluated included body weight (kg), body mass index (BMI, kg/m^2^), protein mass (kg), mineral mass (kg), fat mass (kg and %), skeletal muscle mass (kg and %), lean body mass (kg), fat-free mass (kg), right arm muscle mass (kg), left arm muscle mass (kg), trunk muscle mass (kg), right leg muscle mass (kg), left leg muscle mass (kg), basal metabolic rate (kcal), which was estimated by the InBody 970 device using a proprietary equation based on lean body mass, age, sex, height, and body cell mass, derived from multifrequency bioelectrical impedance analysis, waist-to-hip ratio, total body cell mass (L), skeletal muscle index (SMI), total body water (L), intracellular water (L), extracellular water (L), the extracellular water/total body water ratio, total body water, intracellular water, extracellular water, right arm water content (L), left arm water content (L), trunk water content (L), right leg water content (L), left leg water content (L), total body water/fat-free mass ratio (%), which is a mathematically derived value based on the hydration coefficient (~73%) used to estimate fat-free mass from total body water in BIA, total phase angle (50 kHz), right arm phase angle, left arm phase angle, trunk phase angle, right leg phase angle, and left leg phase angle, among others [32]. The device was calibrated according to the manufacturer’s instructions, and all measurements were conducted by a trained evaluator to ensure consistency and avoid interobserver errors [33].

#### 2.2.3. Hydration Status

Hydration-related variables, including the ECW-to-TBW ratio, were analyzed using BIA parameters. These values were interpreted based on reference norms for athletic populations [34].

All measurements were taken at the same time of day to minimize variability due to hydration or dietary intake. Participants were instructed to abstain from consuming food or fluids for at least three hours before the assessment to avoid fluctuations in impedance readings.

### 2.3. Statistical Analysis

The means and standard deviations were used to describe the study variables by sex. The normality of the data distribution was verified using the Shapiro–Wilk test, while the homogeneity of variances was assessed with Levene’s test. Group differences were analyzed using independent-samples t-tests, and the delta value (∆) between groups was also calculated to indicate absolute mean differences. Effect sizes were assessed through partial eta squared (*η*^2^p), and interpreted as small (<0.01), medium (≥0.059), or large (≥0.138), based on Richardson (2011) [35]. All statistical analyses were performed using JAMOVI^®^ software (version 2.3.21, Sydney, Australia), with a significance level set at *p* < 0.05.

A post hoc statistical power analysis was conducted using G*Power (version 3.1.9.7). Based on the observed difference in ICW between male and female participants (Cohen’s d = 3.96, α = 0.05, and *n*_1_ = 18, *n*_2_ = 16), the achieved statistical power exceeded 0.999.

## 3. Results

Table 2 presents the basic characteristics and body composition of the female participants (*n* = 16) and the male participants (*n* = 18). Men showed significantly greater values than women across nearly all variables, including weight, height, body mass index (BMI), skeletal muscle mass, total lean mass, and fat-free mass (all *p* < 0.001). This difference showed a large effect size (*η*^2^p = 0.784), according to thresholds [35]. In terms of regional muscle distribution, male participants exhibited consistently higher values in upper limbs, lower limbs, and trunk muscle mass. Differences were also observed in basal metabolic rate, total body cell mass, and skeletal muscle index (SMI), with all comparisons favoring male participants and associated with large effect sizes.

Table 3 shows the total body water and regional water distribution of the male (*n* = 18) and female (*n* = 16) participants. Men had significantly greater values for total body water, intracellular water, and extracellular water compared to women, all with large effect sizes. However, the ratio of extracellular water to total body water ECW/TBW did not differ significantly between sexes. Regarding regional water distribution, men showed higher values in all measured segments arms, trunk, and legs while the TBW/FFM% ratio—a derived value based on the fixed hydration coefficient used in the BIA algorithm—was similar between groups, with no significant difference.

Table 4 presents the phase angle values (50 kHz) by body segment for male (*n* = 18) and female (*n* = 16) bodybuilders. Male participants exhibited significantly greater phase angle values in the total body, upper limbs (right and left arms), and trunk, with moderate to large effect sizes (*η*^2^p ≥ 0.257). These differences suggest better cellular integrity and hydration status in men, particularly in the upper body. In contrast, no statistically significant differences were observed between sexes in the lower limbs (right and left legs), where effect sizes were small (*η*^2^p < 0.12).

## 4. Discussion

The present study aimed to analyze and compare body composition and hydration status between male and female elite bodybuilders during an international competition. The findings confirmed the initial hypothesis, revealing significant sex-based differences in intracellular and extracellular water distribution. As expected, male bodybuilders demonstrated a higher proportion of ICW relative to total body water, which corresponds to their greater muscle mass and cell volume. In contrast, female athletes exhibited a higher relative ECW-to-total body water ratio, likely influenced by physiological and hormonal factors that regulate fluid distribution and retention. These results align with previous research linking higher ICW levels to increased muscle hypertrophy and enhanced performance, emphasizing the importance of maintaining an optimal water balance for both aesthetic and functional benefits in bodybuilding. The observed differences between sexes highlight distinct physiological adaptations and underline the need for individualized hydration and training strategies to optimize competition outcomes. Understanding these sex-specific variations can contribute to the development of more precise and evidence-based preparation protocols, ultimately improving performance while minimizing health risks associated with extreme fluid manipulation.

Furthermore, our results are consistent with those of previous studies that have shown that intracellular hydration is an important indicator of muscle health and physical performance. For example, Bosy-Westphal et al. (2017) reported that in trained athletes, a PhA greater than 7.5° was correlated with increased ICW values and increased muscle strength [36]. In comparison, the PhA values found in our study ranged from 7.0 ± 0.9 in females to 8.2 ± 0.7 in males, values that are within the expected range for elite athletes. Kyle et al. (2004) reported that males tend to have a higher ICW/ECW ratio than females do, partly due to hormonal differences and greater total muscle mass [17]. In our study, the mean ICW/ECW ratio was 2.5 ± 0.3 in males and 1.7 ± 0.2 in females, highlighting the consistency of our findings with the literature.

There is also evidence that differences in water distribution may influence training strategies and fluid manipulation prior to competition. For example, previous authors suggested that optimizing ICW through targeted hydration may improve appearance and performance [5,11,37,38]. However, owing to the cross-sectional nature of this study, we did not assess pre- and post-competition variation, so our observations are limited to a single time point. These differences in body composition and water compartments should be interpreted as general characteristics of athletes in a competitive context.

Recent studies also support the use of advanced tools to assess the relationship between intracellular and extracellular water in athletes. For example, Nunes et al. (2022) reported that dietary and hydration strategies implemented the day before competition succeeded in increasing the ICW from 31.6 ± 2.9 L to 33.1 ± 2.8 L, whereas the ECW decreased from 19.8 ± 1.8 L to 17.2 ± 1.4 L, resulting in an increase in the ICW/ECW ratio from 1.60 ± 0.03 to 1.92 ± 0.01 [34] Similarly, Barakat et al. (2022) reported an increase in lean mass and a reduction in subcutaneous thickness by an average of 10%, resulting in significant aesthetic changes during the peak week of preparation [39]. A recent systematic review revealed that near competition, body fat levels are substantially lower, ranging from 8.1 to 18.3% for women and 5.8 to 10.7% for male athletes [40]. The values are within the range of those reported in this study. These findings confirm that strategies focused on intracellular hydration and extracellular water depletion have a direct effect on the appearance and performance of bodybuilders.

Despite the advantages observed in the manipulation of water compartments for optimizing competitive performance, several methodological and physiological limitations must be acknowledged. First, the reliance on bioelectrical impedance analysis (BIA) as the primary assessment tool, although widely validated, presents certain challenges. BIA measurements can be influenced by factors such as hydration status, electrolyte balance, and recent food or fluid intake, potentially affecting the reliability and accuracy of the values obtained [17]. This issue is particularly relevant in the context of bodybuilding competitions, where athletes employ aggressive fluid manipulation strategies that result in rapid fluctuations in body water levels, potentially leading to the misinterpretation of hydration status and body composition metrics. Additionally, variations in body temperature, skin conductivity, and even training-induced muscle inflammation can further impact the precision of BIA readings, making it essential to standardize pre-measurement conditions to minimize inconsistencies. From a physiological perspective, individual differences in hormonal regulation play a significant role in water distribution and retention, introducing additional variability. Hormones such as aldosterone and vasopressin, which regulate sodium balance and water retention, can differ between individuals based on factors like sex, training status, and dietary intake, influencing the intracellular-to-extracellular water ratio and overall hydration balance [5]. These hormonal fluctuations, combined with other factors such as genetic predispositions and metabolic differences, may contribute to inter-individual variability in BIA measurements, potentially limiting the generalizability of the findings to broader bodybuilding populations. Given these limitations, future research should aim to incorporate complementary and more advanced techniques to enhance the accuracy and validity of hydration assessments in bodybuilders. Methods such as magnetic resonance imaging and isotopic dilution techniques could provide more precise evaluations of body water compartments, offering a more comprehensive understanding of hydration dynamics in competitive settings. Additionally, longitudinal studies tracking hydration changes throughout different phases of competition preparation could help establish more reliable baseline values and better inform individualized hydration strategies. By addressing these methodological and physiological limitations, future studies can contribute to the development of more robust and evidence-based guidelines for optimizing hydration and body composition management in bodybuilding.

This study underscores the critical importance of understanding sex-based differences in water compartment distribution among elite bodybuilders and their potential implications for body composition and competitive performance. The findings indicate that male bodybuilders tend to maintain a higher relative proportion of intracellular water ICW to extracellular water ECW, which is strongly associated with enhanced muscle hypertrophy, improved cell function, and a more defined visual appearance. This greater ICW proportion may be attributed to higher skeletal muscle mass, differences in anabolic hormone profiles, and training adaptations specific to male physiology [7]. Conversely, female bodybuilders exhibited a higher relative proportion of ECW, which could be influenced by hormonal fluctuations, particularly related to estrogen levels, as well as differences in fat distribution and connective tissue composition [26,27]. An elevated ECW ratio in females might impact muscle definition and vascularity, key components in competitive bodybuilding aesthetics, and could necessitate specific hydration strategies to mitigate subcutaneous water retention and optimize stage presentation. The distinct ICW and ECW profiles observed between sexes emphasize the need for personalized training, nutrition, and hydration protocols tailored to the physiological characteristics of male and female athletes. These strategies should focus on maximizing muscle fullness through intracellular hydration while carefully managing extracellular fluid to enhance definition without compromising health. Proper hydration management is crucial to prevent potential negative effects of extreme fluid manipulation, such as dehydration, electrolyte imbalances, and impaired muscle function, which can adversely affect both performance and overall well-being [3,15]. Moreover, understanding these differences can aid coaches and sports health professionals in refining peak week (the final week before competition) protocols to achieve optimal balance between muscle size and definition [4]. Considering these findings, future research should further explore the underlying mechanisms driving sex-based differences in fluid distribution, incorporating hormonal profiling and metabolic assessments to develop more comprehensive strategies. Additionally, investigating the impact of different training methodologies and nutritional interventions on ICW and ECW ratios could provide valuable insights to further optimize competitive preparation in both male and female bodybuilders [22]. Ultimately, this study provides a foundation for advancing evidence-based practices in bodybuilding, fostering safer and more effective approaches to achieving peak performance while mitigating health risks associated with improper hydration strategies.

To enhance the practical relevance of our findings, we have expanded the discussion on how these hydration and body composition differences can inform training and dietary strategies for elite bodybuilders. Specifically, we highlight the importance of individualized hydration protocols to optimize muscle definition and performance. By tailoring hydration strategies based on sex-specific physiological characteristics, coaches and athletes can make more informed decisions regarding peak week preparation and overall competition readiness.

### 4.1. Methodological Limitations and Suggestions for Future Research

Although this study provides valuable insights into the relationships between water compartments and body composition in bodybuilders, several limitations must be considered. One of the most significant limitations is the lack of information regarding the specific strategies employed by participants during peak week, a crucial period when athletes implement fluid and carbohydrate manipulation techniques to optimize their stage appearance. Without data on these practices, it is challenging to determine how individualized peak week protocols influenced the observed hydration status and body composition outcomes, which may introduce variability in the results and limit the generalizability of the findings. Future studies should incorporate detailed tracking of peak week interventions, including dietary intake, fluid strategies, and training loads, to better contextualize their effects on hydration and muscle definition.

A key limitation of this study is its cross-sectional design, which provides only a single-time-point snapshot of body composition and hydration status. A longitudinal approach would allow for a more comprehensive understanding of hydration dynamics across different phases of competition preparation. Future research should incorporate repeated measures to track changes in intracellular and extracellular water distribution, as well as the effects of specific training and hydration interventions on competitive outcomes.

Furthermore, the cross-sectional design of this study restricted the ability to assess dynamic changes in water compartments before, during, and after competition. A longitudinal approach would provide a more comprehensive understanding of how fluid manipulation strategies evolve over time and their impact on performance and aesthetics at different stages of preparation. Tracking changes across multiple time points could also help identify the long-term effects of hydration strategies on muscle function and overall health. A significant limitation of this study is the lack of detailed data on participants’ training practices and diet during competition preparation. Additionally, although self-reported data on carbohydrate, protein, and water intake during peak week and prior to weigh-in were collected, the absence of a structured assessment limits a more precise analysis. Given that hydration status and body composition are highly influenced by these factors, future studies should incorporate more systematic evaluations of athletes’ training regimens and nutritional strategies to better understand their impact on hydration, performance, and physique optimization.

Another methodological limitation is the reliance on BIA as the primary assessment tool. Although BIA is widely validated and practical for field use, its accuracy can be compromised by fluid fluctuations, which are common in competitive bodybuilding due to rapid weight-cutting and rehydration strategies. Factors such as electrolyte imbalances, glycogen depletion, and recent dietary intake may affect BIA readings, potentially leading to measurement variability. To enhance accuracy, future research should incorporate complementary techniques such as magnetic resonance imaging or isotopic dilution methods, which offer more precise assessments of body water compartments and tissue composition.

Finally, this study did not account for the influence of key hormonal and metabolic factors, such as aldosterone and vasopressin secretion, which play a critical role in regulating fluid balance and may significantly impact the distribution of intracellular and extracellular water. These factors can vary widely among individuals due to differences in training status, diet, and genetic predisposition, potentially affecting the interpretation of the results. Including hormonal profiling in future studies would provide a more comprehensive understanding of the physiological mechanisms underlying fluid distribution and its impact on bodybuilding performance.

### 4.2. Practical Applications

The findings of this study provide valuable insights for athletes, coaches, and sports nutritionists involved in competitive bodybuilding, emphasizing the critical role of hydration management and individualized strategies in optimizing performance and aesthetics. Understanding the sex-based differences in ICW and ECW water distribution can guide the development of tailored hydration protocols that maximize muscle fullness while minimizing excess subcutaneous fluid, thereby enhancing muscle definition and stage presentation. For male bodybuilders, the greater ICW proportion suggests a focus on strategies that support intracellular hydration, such as optimizing electrolyte balance and carbohydrate loading, to enhance muscle hypertrophy and visual fullness without compromising performance. Conversely, female bodybuilders, with their higher relative ECW levels, may benefit from hydration approaches that help regulate fluid retention and hormonal influences to achieve a leaner, more defined appearance.

Additionally, the results highlight the importance of peak week preparation, where fluid and carbohydrate manipulations can significantly impact body composition. Coaches should closely monitor hydration strategies and avoid excessive fluid restriction or dehydration practices that could impair performance and health. Regular assessment using validated tools, such as BIA, can provide useful real-time feedback on hydration status, although integrating complementary techniques like magnetic resonance imaging or isotopic dilution could further enhance precision in practice. Furthermore, these findings emphasize the need for evidence-based interventions, encouraging athletes to adopt scientific approaches rather than anecdotal or extreme methods that may lead to adverse health effects. Personalized strategies, accounting for factors such as hormonal fluctuations and metabolic variability, can help optimize both competitive results and long-term well-being. By implementing these practical applications, bodybuilding professionals can improve competition preparation, ensuring athletes achieve peak performance with a balanced approach that prioritizes health and sustainability.

## 5. Conclusions

This study successfully characterized and compared body composition and hydration status between male and female elite bodybuilders during an international competition. The findings confirmed the initial hypothesis, demonstrating significant sex-based differences in water compartment distribution. Male bodybuilders exhibited a higher proportion of intracellular water, which is associated with greater muscle mass and cell volume, whereas female athletes presented a higher relative proportion of extracellular water, likely influenced by hormonal and physiological factors. These results highlight the crucial role of intracellular water in optimizing muscle hypertrophy and physical appearance, while the higher extracellular water levels in females may impact their competitive aesthetics and performance strategies.

The insights gained from this study emphasize the importance of individualized hydration and nutrition strategies tailored to the unique physiological characteristics of male and female bodybuilders. The findings provide a scientific basis for developing evidence-based protocols that optimize body composition and performance while minimizing health risks associated with extreme fluid manipulation. Future research should adopt a longitudinal approach to track dynamic changes in hydration status throughout different phases of competition, with a particular focus on peak week interventions to further enhance the understanding of their impact on competitive outcomes.

## Figures and Tables

**Table 1:** Nutrition during the competition week (peak week).

Macronutrient	Male (g/kg/day)	Female (g/kg/day)	Notes
Carbohydrates	3–7 (up to 10 during carb load)	2.5–5.5	Higher intake during carb-loading phase; sometimes up to 10 g/kg
Protein	2.2–3.5	2–2.8	High to preserve lean mass during caloric restriction
Fat	0.5–1	0.5–0.8	Reduced to allow more carbs/protein within caloric limits

**Table 2:** Comparison of anthropometric, body composition, and metabolic variables between male and female bodybuilders.

Descriptives	Females (*n* = 16)		Males (*n* = 18)		∆	*p*-value	*η*^2^p
	Mean	SD	Mean	SD			
Weight (kg)	57.0	5.7	78.0	6.5	21.0	<0.001	0.714
Height (cm)	159.2	6.2	171.3	3.7	12.1	<0.001	0.568
Body Mass Index (kg/m^2^)	22.5	1.6	26.5	1.8	4.0	<0.001	0.522
Fat Mass (kg)	8.1	1.9	7.0	1.7	1,1	0.085	0.094
Fat Mass (%)	14.2	3.0	8.9	1.8	5.3	<0.001	0.498
Musculoskeletal Mass (kg)	27.5	3.1	41.2	3.6	13.7	<0.001	0.781
Musculoskeletal Mass (%)	48.2	2.3	52.8	1.0	4.6	<0.001	0.597
Lean Mass (kg)	46.2	4.8	67.2	5.4	21.0	<0.001	0.789
Fat Free Mass (kg)	48.9	5.0	70.8	5.8	21.9	<0.001	0.783
Right Arm Muscle Mass (kg)	2.6	0.4	4.5	0.4	1.9	<0.001	0.814
Left Arm Muscle Mass (kg)	2.6	0.4	4.5	0.5	1.9	<0.001	0.791
Trunk Muscle Mass (kg)	21.7	2.4	32.3	2.5	10.6	<0.001	0.803
Right Leg Muscle Mass (kg)	7.2	1.0	10.0	0.9	1.8	<0.001	0.655
Left Leg Muscle Mass (kg)	7.2	1.0	9.9	0.9	1.7	<0.001	0.662
Metabolism (kcal)	1.426	110.4	1.903	126.4	477	<0.001	0.782
Waist Hip Ratio	0.788	0.1	0.791	0.1	0.3	0.646	0.007
Total Body Cell Mass	32.4	3.5	47.4	4.0	15.0	<0.001	0.783
Musculoskeletal Index	7.7	0.7	9.8	0.6	2.1	<0.001	0.676

∆ = difference between male and female participants; *p*-value = statistical significance of the comparison between groups (significance level set at *p* < 0.05); and *η*^2^p = partial eta squared, indicating the effect size of the differences, classified as small (<0.01), medium (≥0.059), or large (≥0.138).

**Table 3:** Distribution and comparison of total, intracellular, and extracellular body water between male and female bodybuilders.

Descriptives	Females (*n* = 16)		Males (*n* = 18)		∆	*p*-value	*η*^2^p
	Mean	SD	Mean	SD			
Body Water (L)	35.9	3.7	52.0	4.2	16.1	<0.001	0.784
Intracellular Water (L)	22.6	2.5	33.1	2.8	10.5	<0.001	0.782
Extracellular Water (L)	13.2	1.3	24.5	2.4	11.3	0.069	0.144
ECW/TBW	0.369	0.1	0.363	0.1	0.6	0.180	0.032
TBW	35.9	3.7	52.0	4.2	16.1	<0.001	0.784
ICW	22.6	2.5	33.1	2.8	10.5	<0.001	0.781
ECW	13.2	1.3	18.9	1.5	5.7	<0.001	0.770
Water Right Arm (L)	2.0	0.3	3.5	0.3	1.5	<0.001	0.814
Water Left Arm (L)	2.0	0.3	3.5	0.4	1.5	<0.001	0.791
Water Trunk Arm (L)	16.8	1.8	25.0	1.9	8.2	<0.001	0.803
Water Right Leg (L)	5.5	0.8	7.7	0.7	2.2	<0.001	0.670
Water Left Leg (L)	5.5	0.7	7.7	0.7	2.2	<0.001	0.666
TBW/FFM (%)	73.3	0.2	73.3	0.3	0.0	0.502	0.006

∆ = difference between male and female participants; *p*-value = statistical significance of the comparison between groups (significance level set at *p* < 0.05); *η*^2^p = partial eta squared, indicating the effect size of the differences, classified as small (<0.01), medium (≥0.059), or large (≥0.138); ECW/TBW = ratio of extracellular water to total body water; TBW = total body water; ICW = intracellular water; ECW = extracellular water; and TBW/FFM (%) = percentage of total body water relative to fat-free mass.

**Table 4:** Comparison of angular phases by body segment between male and female bodybuilders.

Descriptives	Female (*n* = 16)		Male (*n* = 18)		∆	*p*-value	*η*^2^p
	Mean	SD	Mean	SD			
Total angular phase (50 Khz)	7.0	0.9	8.2	0.7	1.2	0.003	0.257
Angular phase of the right arm (50 Khz)	6.9	0.8	8.0	0.7	1.1	<0.001	0.337
Angular phase of the left arm (50 Khz)	6.7	0.9	7.9	0.6	1.2	<0.001	0.330
Angular phase of the trunk (50 Khz)	10.3	1.5	12.9	1.5	2.6	<0.001	0.357
Angular phase of the right leg (50 Khz)	7.0	1.1	8.5	0.8	1.5	0.005	0.116
Angular phase of the left leg (50 Khz)	6.9	1.2	7.9	0.9	1.0	0.007	0.094

∆ = difference between male and female participants; *p*-value = statistical significance of the comparison between groups (significance level set at *p* < 0.05); and *η*^2^p = Partial eta squared, indicating the effect size of the differences, classified as small (<0.01), medium (≥0.059), or large (≥0.138).

## Data Availability

The data can be requested from the corresponding author.

## References

[1] Aragon A.A., Schoenfeld B.J., Wildman R., Kleiner S., VanDusseldorp T., Taylor L., Earnest C.P., Arciero P.J., Wilborn C., Kalman D.S. (2017). International Society of Sports Nutrition Position Stand: Diets and Body Composition. J. Int. Soc. Sports Nutr..

[2] Olshvang D., Harris C., Chellappa R., Santhanam P. (2024). Predictive Modeling of Lean Body Mass, Appendicular Lean Mass, and Appendicular Skeletal Muscle Mass Using Machine Learning Techniques: A Comprehensive Analysis Utilizing NHANES Data and the Look Ahead Study. PLoS ONE.

[3] Gann J.J., Andre T.L., Gallucci A.R., Willoughby D.S. (2021). Effects of Hypohydration on Muscular Strength, Endurance, and Power in Women. J. Strength Cond. Res..

[4] Escalante G., Stevenson S.W., Barakat C., Aragon A.A., Schoenfeld B.J. (2021). Peak Week Recommendations for Bodybuilders: An Evidence-Based Approach. BMC Sports Sci. Med. Rehabil..

[5] Maughan R.J., Shirreffs S.M. (2010). Development of Hydration Strategies to Optimize Performance for Athletes in High-Intensity Sports and in Sports with Repeated Intense Efforts. Scand. J. Med. Sci. Sports.

[6] Casa D.J., Stearns R.L., Lopez R.M., Ganio M.S., McDermott B.P., Yeargin S.W., Yamamoto L.M., Mazerolle S.M., Roti M.W., Armstrong L.E. (2010). Influence of Hydration on Physiological Function and Performance During Trail Running in the Heat. J. Athl. Train..

[7] Schoenfeld B.J., Alto A., Grgic J., Tinsley G., Haun C.T., Campbell B.I., Escalante G., Sonmez G.T., Cote G., Francis A. (2020). Alterations in Body Composition, Resting Metabolic Rate, Muscular Strength, and Eating Behavior in Response to Natural Bodybuilding Competition Preparation: A Case Study. J. Strength Cond. Res..

[8] Cataldi D. (2023). Methodological Considerations of Body Composition Assessments and Predicting Athletic Performance: The Da Kine Study. Ph.D. Thesis.

[9] Homer K.A., Cross M.R., Helms E.R. (2024). Peak Week Carbohydrate Manipulation Practices in Physique Athletes: A Narrative Review. Sports Med.-Open.

[10] Cheuvront S.N., Kenefick R.W. (2011). Dehydration: Physiology, Assessment, and Performance Effects. Compr. Physiol..

[11] Martín-Rodríguez A., Belinchón-deMiguel P., Rubio-Zarapuz A., Tornero-Aguilera J.F., Martínez-Guardado I., Villanueva-Tobaldo C.V., Clemente-Suárez V.J. (2024). Advances in Understanding the Interplay between Dietary Practices, Body Composition, and Sports Performance in Athletes. Nutrients.

[12] Petri C., Micheli M.L., Izzicupo P., Timperanza N., Lastrucci T., Vanni D., Gulisano M., Mascherini G. (2023). Bioimpedance patterns and bioelectrical impedance vector analysis (BIVA) of body builders. Nutrients.

[13] Lebiedowska A., Hartman-Petrycka M., Stolecka-Warzecha A., Odrzywołek W., Bożek M., Wilczyński S. (2024). The Influence of Skin Parameters and Body Composition on the Tolerance of Pain Stimulus Generated During Electrical Muscle Stimulation (EMS) in Women-Pilot Study. Clin. Cosmet. Investig. Dermatol..

[14] Montain S.J., Latzka W.A., Sawka M.N. (1999). Fluid Replacement Recommendations for Training in Hot Weather. Mil. Med..

[15] Wilson P.B. (2021). Associations of Urine Specific Gravity with Body Mass Index and Lean Body Mass at the Population Level: Implications for Hydration Monitoring. Int. J. Sport Nutr. Exerc. Metab..

[16] Aburto-Corona J.A., Calleja-Núñez J.J., Moncada-Jiménez J., de Paz J.A. (2024). The Effect of Passive Dehydration on Phase Angle and Body Composition: A Bioelectrical Impedance Analysis. Nutrients.

[17] Kyle U.G., Bosaeus I., De Lorenzo A.D., Deurenberg P., Elia M., Gómez J.M., Lilienthal Heitmann B., Kent-Smith L., Melchior J.C., Pirlich M. (2004). Bioelectrical Impedance Analysis—Part I: Review of Principles and Methods. Clin. Nutr..

[18] Di Vincenzo O., Marra M., Di Gregorio A., Pasanisi F., Scalfi L. (2021). Bioelectrical Impedance Analysis (BIA)-Derived Phase Angle in Sarcopenia: A Systematic Review. Clin. Nutr..

[19] Matias C.N., Campa F., Nunes C.L., Francisco R., Jesus F., Cardoso M., Valamatos M.J., Mil Homens P., Sardinha L.B., Martins P. (2021). Phase Angle Is a Marker of Muscle Quantity and Strength in Overweight/Obese Former Athletes. Int. J. Environ. Res. Public Health.

[20] Short T., Yamada P. (2024). Exploring the Mechanistic Trail Connecting Cellular Function, Health, and Athletic Performance with Phase Angle: A Review on the Physiology of Phase Angle and Exercise-Based Interventions. Top. Exerc. Sci. Kinesiol..

[21] Catapano A., Trinchese G., Cimmino F., Petrella L., D’Angelo M., Di Maio G., Crispino M., Cavaliere G., Monda M., Mollica M.P. (2023). Impedance Analysis to Evaluate Nutritional Status in Physiological and Pathological Conditions. Nutrients.

[22] Almeida F.N., Nascimento D.C., Moura R.F., Peixoto D.L., Moraes W.M.A.M., Schoenfeld B.J., de Sousa Neto I.V., Prestes J. (2023). Training, Pharmacological Ergogenic Aids, Dehydration, and Nutrition Strategies During a Peak Week in Competitive Brazilian Bodybuilders: An Observational Cross-Sectional Study in a Non-World Anti-Doping Agency Competitive Environment. Sports.

[23] Campa F., Gobbo L.A., Stagi S., Cyrino L.T., Toselli S., Marini E., Coratella G. (2022). Bioelectrical Impedance Analysis Versus Reference Methods in the Assessment of Body Composition in Athletes. Eur. J. Appl. Physiol..

[24] Lombardo M., Feraco A., Armani A., Camajani E., Gorini S., Strollo R., Padua E., Caprio M., Bellia A. (2024). Gender Differences in Body Composition, Dietary Patterns, and Physical Activity: Insights from a Cross-Sectional Study. Front. Nutr..

[25] Lukaski H., Raymond-Pope C.J. (2021). New Frontiers of Body Composition in Sport. Int. J. Sports Med..

[26] Pasiakos S.M., McClung H.L., Margolis L.M., Murphy N.E., Lin G.G., Hydren J.R., Young A.J. (2015). Human Muscle Protein Synthetic Responses During Weight-Bearing and Non-Weight-Bearing Exercise: A Comparative Study of Exercise Modes and Recovery Nutrition. PLoS ONE.

[27] Macho J., Mudrak J., Slepicka P. (2021). Enhancing the Self: Amateur Bodybuilders Making Sense of Experiences with Appearance and Performance-Enhancing Drugs. Front. Psychol..

[28] Taniguchi M., Yamada Y., Yagi M., Nakai R., Tateuchi H., Ichihashi N. (2021). Estimating Thigh Skeletal Muscle Volume Using Multi-Frequency Segmental-Bioelectrical Impedance Analysis. J. Physiol. Anthropol..

[29] World Medical Association (2013). World Medical Association Declaration of Helsinki: Ethical Principles for Medical Research Involving Human Subjects. JAMA.

[30] Yi Y., Baek J.Y., Lee E., Jung H.-W., Jang I.-Y. (2022). A Comparative Study of High-Frequency Bioelectrical Impedance Analysis and Dual-Energy X-Ray Absorptiometry for Estimating Body Composition. Life.

[31] Jackson A.S., Pollock M.L., Graves J.E., Mahar M.T. (1988). Reliability and Validity of Bioelectrical Impedance in Determining Body Composition. J. Appl. Physiol..

[32] Ha Y.-C., Kim S., Yoo J.-I. (2024). Open, Active-Controlled Clinical Study to Evaluate the Correlation Between Whole Body DEXA and BIA Muscle Measurements. J. Bone Metab..

[33] McLester C.N., Nickerson B.S., Kliszczewicz B.M., McLester J.R. (2020). Reliability and Agreement of Various InBody Body Composition Analyzers as Compared to Dual-Energy X-Ray Absorptiometry in Healthy Men and Women. J. Clin. Densitom..

[34] Nunes J.P., Araújo J.P., Ribeiro A.S., Campa F., Schoenfeld B.J., Cyrino E.S., Trindade M.C. (2022). Changes in Intra-to-Extra-Cellular Water Ratio and Bioelectrical Parameters from Day-before to Day-of Competition in Bodybuilders: A Pilot Study. Sports.

[35] Richardson J.T.E. (2011). Eta Squared and Partial Eta Squared as Measures of Effect Size in Educational Research. Educ. Res. Rev..

[36] Bosy-Westphal A., Jensen B., Braun W., Pourhassan M., Gallagher D., Müller M.J. (2017). Quantification of Whole-Body and Segmental Skeletal Muscle Mass Using Phase-Sensitive 8-Electrode Medical Bioelectrical Impedance Devices. Eur. J. Clin. Nutr..

[37] Ramos Campo D.J., Martínez Sánchez F., Esteban García P., Rubio Arias J.Á., Bores Cerezal A., Clemente Suárez V., Jiménez Díaz J.F. (2014). Body Composition Features in Different Playing Position of Professional Team Indoor Players. Int. J. Morphol..

[38] Belinchon-deMiguel P., Clemente-Suárez V.J. (2018). Psychophysiological, Body Composition, Biomechanical and Autonomic Modulation Analysis Procedures in an Ultraendurance Mountain Race. J. Med. Syst..

[39] Barakat C., Escalante G., Stevenson S.W., Bradshaw J.T., Barsuhn A., Tinsley G.M., Walters J. (2022). Can Bodybuilding Peak Week Manipulations Favorably Affect Muscle Size, Subcutaneous Thickness, and Related Body Composition Variables? A Case Study. Sports.

[40] Bauer P., Majisik A., Mitter B., Csapo R., Tschan H., Hume P., Martínez-Rodríguez A., Makivic B. (2023). Body Composition of Competitive Bodybuilders: A Systematic Review of Published Data and Recommendations for Future Work. J. Strength Cond. Res..

